# Kinetic control of nucleosome displacement by ISWI/ACF chromatin remodelers

**DOI:** 10.1186/1756-8935-6-S1-P7

**Published:** 2013-03-18

**Authors:** Ana-Maria Florescu, Helmut Schiessel, Ralf Blossey

**Affiliations:** 1 Max-Planck-Institute for the Physics of Complex Systems, Nöthnitzer Strasse 38, D-01187 Dresden, Germany; 2 Interdisciplinary Research Institute, Université des Sciences et des Technologies de Lille (USTL), CNRS USR 3078,50, Avenue Halley, 59568 Villeneuve d’Ascq, France; 3 Lorentz Institut voor de theoretische natuurkunde, Universiteit Leiden, P.O. Box 9506, NL-2300 RA Leiden, The Netherlands

## 

Chromatin structure is dynamically organized by chromatin remodelers, motor protein complexes which move and remove nucleosomes. The regulation of remodeler action has recently been proposed to underlie a kinetic proofreading scheme which combines the recognition of histone-tail states and the ATP-dependent loosening of DNA around nucleosomes. Members of the ISWI-family of remodelers additionally recognize linker length between nucleosomes. Here, we show that the additional proofreading step involving linker length alone is sufficient to promote the formation of regular arrays of nucleosomes. ATP-dependent remodeling by bidirectional motors is shown to reinforce positioning as compared to statistical positioning.

